# Digital health in pharmacy education: Elective practical course integrating wearable devices and their generated health data

**DOI:** 10.1016/j.rcsop.2024.100465

**Published:** 2024-06-11

**Authors:** Florian Kinny, Sabina Schlottau, Bushra Ali Sherazi, Emina Obarcanin, Stephanie Läer

**Affiliations:** a Heinrich-Heine University Duesseldorf, Institute of Clinical Pharmacy and Pharmacotherapy, Universitaetsstr. 1, 40225 Duesseldorf, Germany; b Institute of Pharmacy, Faculty of Pharmaceutical and Allied Health Sciences, Lahore College for Women University, Lahore 54000, Pakistan; c Lee Kong Chian School of Medicine, Nanyang Technological University, Singapore

**Keywords:** mHealth, Wearables, CGM, Pharmaceutical care, Education

## Abstract

The widespread adoption of wearable devices (wearables) for monitoring vital signs, including blood pressure and glucose levels, has experienced a considerable surge in recent times. This surge has led to the generation of a substantial amount of health data, accessible to pharmacists during patient consultations as the healthcare sector advances in digitalization. To enhance the digital competencies of future pharmacists required by the rapidly changing digital health landscape, the Institute of Clinical Pharmacy and Pharmacotherapy, Heinrich Heine University (HHU) Duesseldorf has developed an innovative elective practical course aimed to bolster pharmacy students' competencies in handling wearables and the health data generated. The three-week practical elective course employed wearables FreeStyle Libre® 3 (Continuous Glucose Monitoring, CGM) and Aktiia (Cuffless Blood Pressure Monitoring). The hands-on activities allowed participants to obtain and interpret wearable-generated health-related data and acquainted them with simulated patient cases. Final-year pharmacy students' subjective assessments before and after the course depicted the increased knowledge and competence regarding analysing wearables data.

## 1 Introduction

Digital health interventions have revolutionized healthcare, as evidenced by wearable technology with the ability to monitor markers of health status and predict health events.^1^ Wearable devices, referred to as digital tools, can continuously monitor vital signs and provide a comprehensive view of a patient's clinical condition or therapy.^2^ For instance, the minimal invasive, transcutaneous systems that measure interstitial glucose levels continuously each minute have been reported to have some clinical advantage over self-monitoring of blood glucose (SMBG).^3^ Today's digital sensors can conveniently provide real-time glucose data to help making informed decisions about treatment and lifestyle.^4^ Developments in blood pressure monitors are also moving towards continuous management to enable comfortable measurement even during the night. The Aktiia bracelet uses optical signals (photoplethysmography, PPG) to determine blood pressure through pulse wave analysis.^5^ Such digital tools for continuous glucose and blood pressure monitoring are commercially available and described in the literature.^6^^,^^7^ In Germany, the real-time CGM systems have been reimbursed by statutory health insurance companies for insulin-dependent diabetes patients since 2016.^8^

The widespread adoption of wearables, by individuals for lifestyle monitoring purposes, has resulted in the generation of a considerable amount of data.^9^ In addition, there is a growing use of mobile health (mHealth) tools among patients to prevent and manage chronic diseases with evidence of non-inferiority over traditional care.^10^ Healthcare professionals are also supporting their patients through these technologies worldwide.^11^ Among others, pharmacists contribute significantly to improving clinical outcomes by providing direct patient care using new diabetes technology.^12^ While pharmacists, being easily accessible and highly qualified healthcare professionals, will undoubtedly encounter and engage with wearables and their valuable data, they may lack the necessary skills and training to do this effectively.^13^

Since the need for digital health literacy has increased in times of advancing digitalization in healthcare,^14^ the integration of courses for pharmacists on digital topics is essential. To meet this educational need and to adapt to this evolving landscape of digital health competencies for future pharmacists, we developed a practical elective course at the Institute of Clinical Pharmacy and Pharmacotherapy, Heinrich Heine University, Duesseldorf, Germany for final-year pharmacy students. Through this pilot project, we wanted to explore the feasibility of such an elective practical course for student engagement and future implications.

## 2 Methods

### 2.1 Course Design, Implementation and procedures

**Fig. 1:**
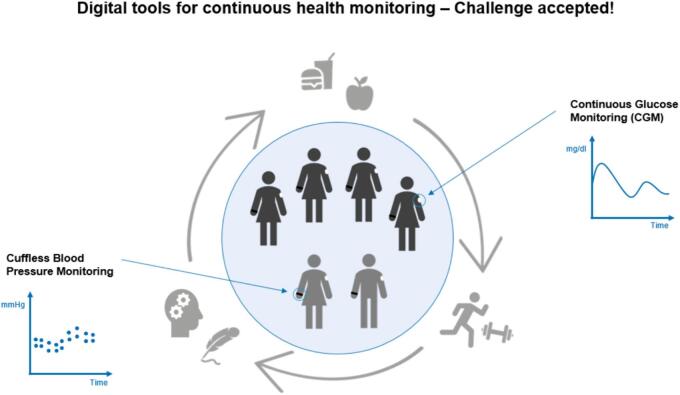
Design and Implementation of the Practical Elective Course.

As the course involved the collection of personal health data in real time, ethical approval was obtained from the responsible ethics committee of medical faculty of Heinrich-Heine-University Duesseldorf (Number: 2023–2409) and all the participants signed informed consent before the start of the course. The three-week course was conducted in August 2023 as part of the graduate pharmacy education program. The overall design of the elective practical course is displayed in Fig. 1.

**Fig. 2:**
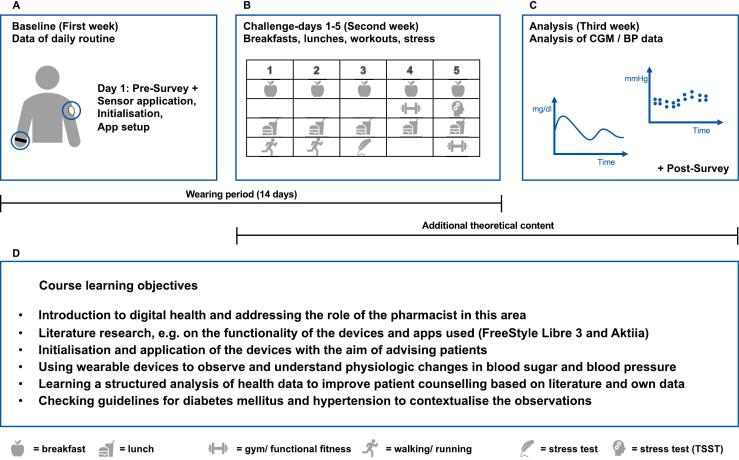
Timeline and course content. The course can be divided into three components (**A-C**): **A** On the first day, the students answered the pre-survey and then applied the sensors. Glucose and blood pressure values were measured for one week without any challenges (baseline). **B** During the second week, various food, workout and stress challenges were carried out. Theoretical content (such as guidelines and scientific literature) was also discussed. **C** The sensors were removed after the second week. In addition to theoretical content, the third week mainly involved analysing the data collected. **D** This section contains an overview of the course learning objectives. CGM: Continuous Glucose Monitoring, BP: Blood Pressure.

The course consisted of didactic content about digital health and mHealth tools as well as a unique hands-on experience with two specific wearables, focussing on their functionality and practical applications. After enrolment and the informed consent process, the students were tasked with initiating and calibrating the devices themselves. The course utilized the CGM device FreeStyle Libre 3 (Abbott GmbH, Germany) and the blood pressure monitoring bracelet Aktiia (Aktiia SA, Switzerland). Each participant wore both devices simultaneously for 14 days. During this period, participants followed standardized eating patterns with breakfast and lunch on five consecutive days (dinner not included) to understand the impact of different foods, their quantities, and compositions on different physiological parameters such as glucose levels and blood pressure. The food characteristics are depicted in supplementary material A (S1, Table A1). Additionally, they engaged in four different sports sessions of varying intensities to investigate the effects of physical activity on vital signs. These sessions included brisk walking for 45 min, 5 km-jogging, exercises with weights in a gym for 40 min and functional-fitness exercises for 40 min (S1, Table A2). To examine the impact of stress on blood pressure and glucose levels, students underwent two tasks: 1) an exam on general knowledge, consisting of 20 blocks with a time limit of three minutes per block (S1, Table A3), and 2) a modified version of the Trier Social Stress Test (TSST),^15^ focusing solely on public speaking in which they had to present unfamiliar slides in front of a committee comprising three individuals. The course content and timeline are summarized in Fig. 2.

Over the 14-day device-wearing period, the participants familiarized themselves with the associated software and apps, allowing them to record information such as meals, illnesses, and physical activities. They learned how to interpret data displayed on the devices. Students gained practical experience in data analysis and interpretation using clinical patient cases. They were supervised by two faculty members who also wore the digital tools. To assess the effectiveness of this course, pre- and post-surveys were administered to the participants. The participants were asked to rate the statements in the surveys using a Likert Scale consisting of seven possible answers (1 = “very strongly disagree”, 2 = “strongly disagree”, 3 = “disagree”, 4 = “neither agreeing nor disagreeing”, 5 = “agree”, 6 = “strongly agree”, 7 = “very strongly agree”) (S2, Table A4). This self-assessment allowed them to evaluate their skills and knowledge about wearables in general, as well as their ability to provide consultation based on the data collected from these devices. Descriptive statistics were used to analyse survey data.

## 3 Results

Four final-year pharmacy students participated in the elective practical course. The innovative practical course helped the participants strengthen their digital health skills and prepared them for future digital health challenges as depicted by their subjective assessments before and after the course (Table 1). The hands-on activities provided participants with opportunities to obtain and interpret wearable-generated health-related data and acquainted them with simulated patient cases.

**Table 1:** Participant Characteristics and pre-post-course survey responses (*N* = 4).

Age	23.50 ± 2.12 (Mean ± SD)	
Gender	Female (100%)	
Knowledge and competence regarding CGM		
Items	**Pre-Survey scores (Mean ± SD)**	**Post-Survey scores (Mean ± SD)**
I know the indications for CGM systems.	4.50 (± 0.58)	6.25 (± 0.50)
I am familiar with the structure of a CGM sensor.	3.25 (± 1.71)	5.25 (± 1.50)
I feel competent enough to apply a CGM system	2.50 (± 1.29)	6.75 (± 0.50)
I know how a CGM sensor performs a glucose measurement	4.50 (± 0.58)	6.50 (± 0.58)
I can interpret the CGM health metrics	2.00 (± 1.15)	5.75 (± 0.50)
I feel competent enough to interpret CGM data from people with diabetes	2.25 (± 1.50)	6.00 (± 0.82)
I know the effect of different carbohydrates on the glucose curve	2.75 (± 0.96)	5.75 (± 0.96)
The advantages of a CGM device over SMBG are clear to me	3.50 (± 1.91)	5.75 (± 1.26)
I feel competent enough to advise people with diabetes on the use and operation of a CGM system	2.50 (± 1.29)	6.50 (± 0.58)
I feel competent enough to make treatment recommendations based on CGM data	2.50 (± 1.91)	5.50 (± 1.00)
Knowledge and competence regarding blood pressure monitoring		
I know the differences between various blood pressure measurement methods.	3.75 (± 1.71)	5.75 (± 0.96)
I can explain the 24-h blood pressure measurement.	2.00 (± 1.15)	5.50 (± 1.29)
I am familiar with photoplethysmography (PPG) as a technology.	1.25 (± 0.50)	6.00 (± 0.82)
I am familiar with the blood pressure limits for the diagnosis of arterial hypertension.	4.25 (± 2.22)	6.25 (± 0.96)
I can explain the relevance of arterial hypertension in patients with type 2 diabetes mellitus.	4.75 (± 2.22)	6.25 (± 0.50)
I know the contents of the current guidelines on arterial hypertension and type 2 diabetes mellitus.	5.00 (± 0.82)	6.00 (± 0.82)
I have already measured my own blood pressure.	5.25 (± 2.36)	6.25 (± 0.96)
I feel competent enough to provide patient with comprehensive advice on the subject of blood pressure.	3.25 (± 1.89)	5.75 (± 0.50)
I feel qualified to educate patients regarding their blood pressure values.	4.00 (± 1.41)	5.75 (± 0.50)
I feel qualified to advise polypharmacised patients with type 2 diabetes mellitus and hypertension.	3.00 (± 1.63)	5.00 (± 0.82)
Post-Satisfaction Survey		
Taking part in the study was an added value for me personally.	**–**	5.80 (± 0.50)
I think continuous monitoring of vital signs in patients makes sense.	**–**	5.80 (± 0.50)
I think continuous monitoring of vital signs in healthy people makes sense.	**–**	5.50 (± 0.60)
Using a blood pressure monitor and a CGM system has increased my digital competence	**–**	5.50 (± 0.60)

SD = Standard Deviation, CGM = Continuous Glucose Monitoring, SMBG = Self-Monitoring Blood Glucose (1 = “very strongly disagree”, 2 = “strongly disagree”, 3 = “disagree”, 4 = “neither agreeing nor disagreeing”, 5 = “agree”, 6 = “strongly agree”, 7 = “very strongly agree”).

**Fig. 3:**
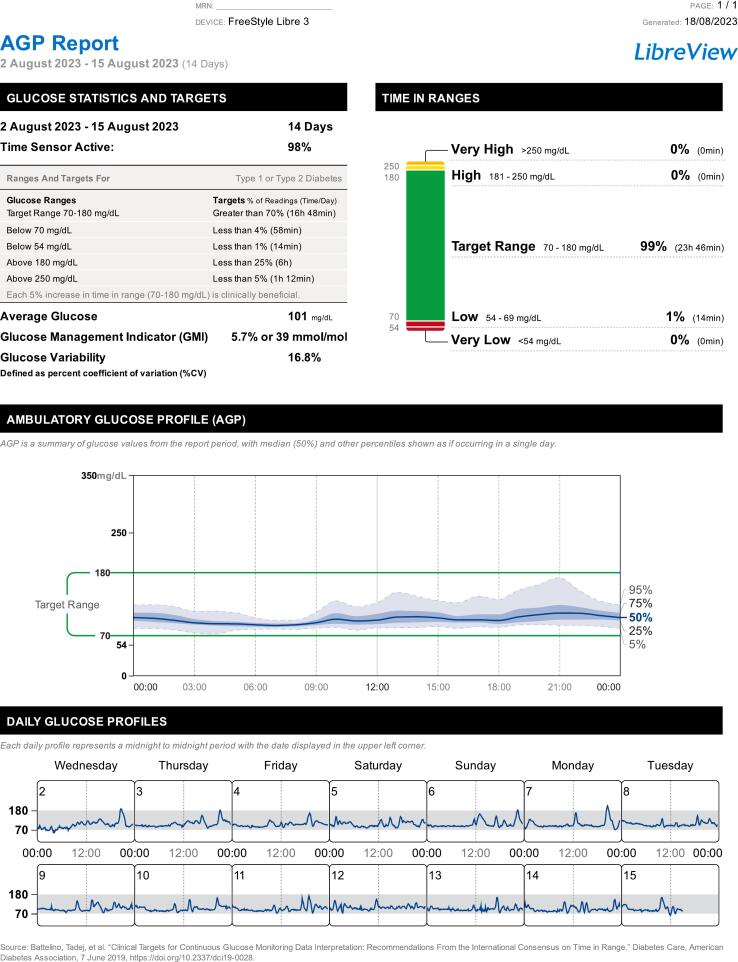
Anonymous AGP-Report (August 2023). The AGP-Report is a standardized, single-page report that summarises glucose data assessed by continuous glucose monitor devices. It includes wear statistics such as the wear period and time the sensor was active as well as glucose statistics such as the average glucose value, glucose management indicator and glucose variability defined as coefficient of variance in %, both displayed in the top left corner. In the top right corner, the time in specific ranges is displayed. The amount of the time of the wear period in each range is shown in a stacked bar chart. In the middle of the report the AGP is laid out as a summary of glucose values from the report period, with median, inter-quartile-range and 5–95-percentile shown as if occurring in a single day. On the bottom of the report each daily glucose profile (midnight to midnight) is shown. AGP: Ambulatory Glucose Profile.

**Fig. 4:**
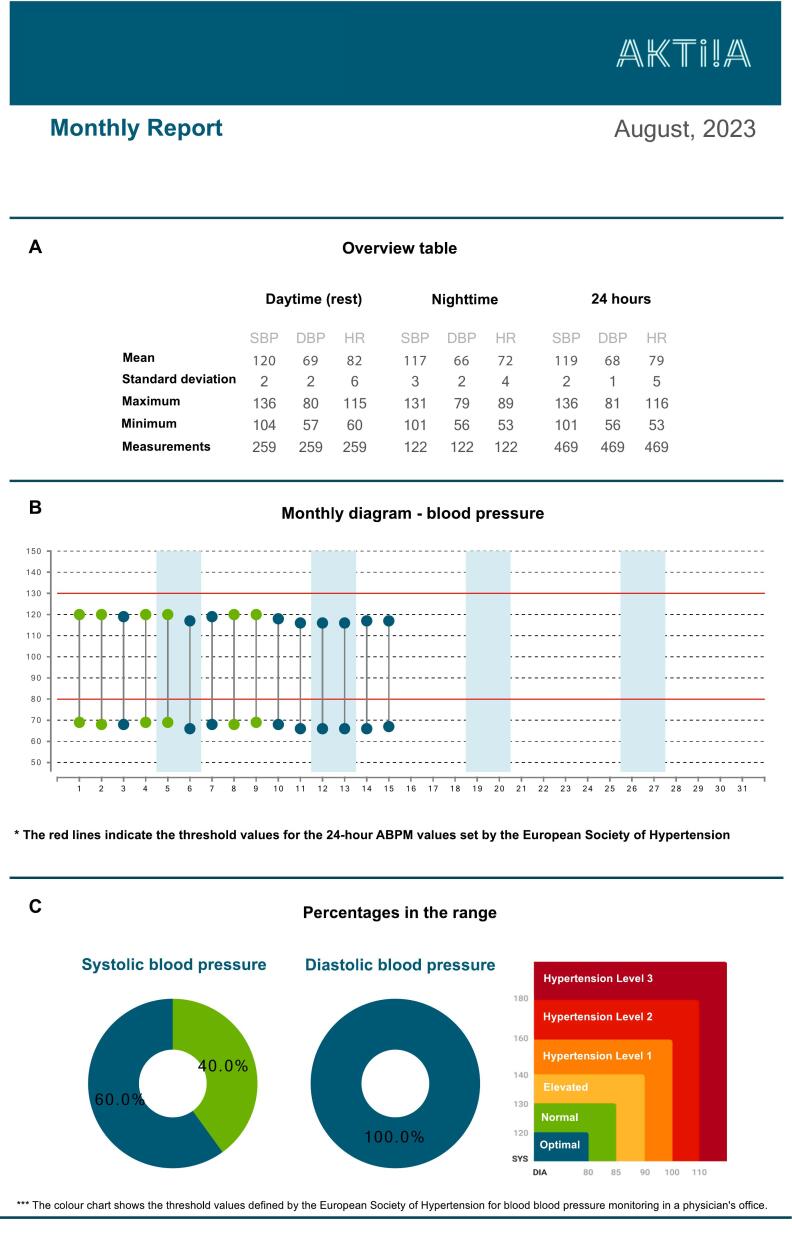
Anonymous Aktiia blood pressure report (August 2023). Modified to English language. The report consists of three sections **(A-C)**: **A** Overview table with mean values, standard deviation, maximum, minimum and number of measurements for daytime, night time and 24 h. SBP: systolic blood pressure, DBP: diastolic blood pressure, HR: heart rate. **B** Monthly diagram for blood pressure. The upper dots represent the mean systolic blood pressure for each day and the lower dots represent the mean diastolic blood pressure for each day. The red lines indicate the recommended threshold values for the 24-h-blood-pressure-measurement values by the European Society of Hypertension.^16^ The colour of the dots correlates with the blood pressure classification (see C). APBM: Ambulatory Blood Pressure Monitoring. **C** This section shows the percentages in the range for systolic and diastolic blood pressure. The ranges correspond to the blood pressure classification of the European Society of Hypertension.^16^ For example, petrol colour stands for <120/80 mmHg (optimal) and green colour stands for 120–129/80–84 mmHg (normal). (For interpretation of the references to colour in this figure legend, the reader is referred to the web version of this article.)

The main focus was on CGM sensors and blood pressure monitoring bracelets. Students learned how the measurements were conducted and how to operate and initialize the devices. The CGM system measured continuously the glucose levels in the interstitial fluid every minute. The app corresponding to the CGM displayed the current data in various graphs and categorised the data according to a colour scheme. The diary, statistics, and reporting functions integrated into the software were also used and assessed weekly (Fig. 3, Fig. 4).

After the 14-day measurement period, it was possible to export the CGM-data as an ambulatory glucose profile (AGP) and blood pressure data as a blood pressure report (Fig. 3, Fig. 4). The students felt confident on how to approach the analysis systematically to enhance patient care and counselling.

The Aktiia blood pressure bracelet took an average of 33 measurements of pulse, systolic and diastolic blood pressure per day. Measurements were taken automatically throughout day and night, with the exception when the person was in motion. These measurements resulted in a 24-h blood pressure profile. The Aktiia app provides a report (Fig. 4) that shows not only the individual measurements but also average values for day and night as well as a classification of the values according to the European Society of Hypertension guidelines.^16^

In general, the digital tools were well accepted. The students generally had no problems initializing the devices and connecting them to a mobile device. As seen in Table 1 students gained knowledge and confidence from the course. According to the results of the survey, they felt able to make recommendations based on what they had learned and felt competent enough to advise on wearables. The students stated that they were very satisfied with this course, and using the CGM system and blood pressure monitor with associated software and mobile applications increased their digital health competencies (Table 1). Informal feedback at the end of the course indicated that students enjoyed attending this course and acquiring their own personal health-related data through wearables motivated them for setting their own health goals.

## 4 Discussion

The elective practical course on wearables and the health data generated provided guidance on wearables, such as CGM systems and blood pressure monitors, including their operation and functionality to the pharmacy students. It allowed them to enhance their digital health knowledge and skills. The practical activities further encouraged students to apply the knowledge and skills they have gained into the process of pharmaceutical care, e.g., patient consultation. In addition, students reported that practical elective course improved their understanding and knowledge of wearable technologies and their integration into future professional practice.

In the ever-changing digital healthcare landscape, it is an imperative to prepare pharmacists and pharmacy students for digital health technology challenges. Increasing adoption of digital health interventions in pharmacy practice requires a digitally literate pharmacy workforce.^17^ However, the International Pharmaceutical Federation indicated in its global survey on “digital health in pharmacy education” that very few pharmacy schools have integrated digital health education into their curricula.^13^ The existing educational approaches included theoretical and experiential learning as well as simulations involving digital technologies.^17^ The design of our practical elective course allowed pharmacy students to get theoretical insights into wearable technology as well as novel experiential learning involving the effect of food, physical activity, and stress on physiological parameters. Thus, the results of the elective practical course confirm previous studies which demonstrated that the experience of wearing digital devices and interpreting health-related data enhance engagement and learning among medical, dental, and pharmacy students, as well as practicing pharmacists.^18^^,^^19^ Wearables have become increasingly popular in recent years for fitness, preventive health care, and chronic disease monitoring.^20^ Pharmacists being more frequently visited by patients than other healthcare professionals^21^ are best positioned to address patient needs regarding their wearable devices and generated data. We believe that this course has laid an important foundation in this regard, as the pharmacy curriculum needs to be adapted to include more digital health content in light of the technological advancements in healthcare.

We acknowledge some limitations to our pilot project. Firstly, a small number of course participants may limit the generalizability of our results. However, a maximum number of four participants could enrol in such an elective course according to the university policies. Secondly, the qualitative survey data was based on self-reported subjective assessments of students. We did not measure any objective skill competencies at the end of the course as they were beyond the scope of this project. Future courses focussing on wearables could include sensors for continuous monitoring of other health markers and more robust patient care scenarios that could help the next generation of pharmacists to better prepare for future healthcare challenges whether routine or emergent.

To our knowledge, this is the first course in Germany in which pharmacy students have been able to strengthen their digital skills in the field of diabetes mellitus and hypertension by generating and interpreting their own vital signs. However, this also raises the question of logistics where a large number of students need to be enrolled. There are examples in the literature where such courses were conducted with larger groups of students. For instance, Sherrill et al. conducted a two-week CGM practice module in the United States (US) with 37 pharmacy students and 6 pharmacists that resulted in increased CGM knowledge and confidence.^19^ Similarly, Ward et al. developed the ‘Wearables in Healthcare’ course in the US, with 21 biomedical engineering students wearing a medical-grade wrist sensor throughout the 16-week semester. The 21 students were provided access to the raw data so that they could analyse their own data. The focus of this study was more on data analysis than would be necessary with pharmacy students. However, the authors describe some considerations for implementing a similar course: Students have different levels of prior knowledge; there may be different attitudes towards data privacy; misunderstanding of personal physiological data may lead to health concerns; an increase in group size should be achieved without compromising individual attention; and the affordability of the wearables.^22^ The following conclusions can be drawn for a possible implementation of a wearable learning module in the pharmacy degree programme:

• Wearing a wearable needs to be on a voluntary basis. Participation in the digital health seminar must also be guaranteed for students with concerns of any kind.
• The wearables need to be financed by the university/through a cooperation.
• Sufficient staff needs to be available to provide students with sufficient support. The number of staff is increased by the fact that individual values may require individual clarification by the staff.
• The time required must be considered. For the students, installing, carrying and, if necessary, noting down activities requires more time than is usually estimated for a seminar. Supervisors need sufficient time to prepare teaching materials, impart knowledge about wearables and prepare the data.

The future pharmacists in our course have indicated that the elective, practical course has provided them with personal added value. When asked, all of the biomedical engineering students responded that they would recommend the course to other students.^22^ In 2020, 16 medical faculties at German universities reported teaching digital health content, either as an elective or compulsory course, which supports that implementation is possible despite deployment challenges.^23^ In summary, there is an urgent need in Germany to educate pharmacy students in digital health contents similar to medical students.

## 5 Conclusion

The increased availability and usage of wearables require pharmacy students to be prepared for related patient needs. The elective practical course on wearables and the health data they generate will enable future pharmacists to provide wearable counselling and guidance on wearables, such as CGM systems and blood pressure monitors, including their operation and functionality. Moreover, the interpretation of health data and subsequent recommendations for action will also enhance clinical skills and experience. Based upon our pilot project, a digital health course with practical elements appears to be feasible, provided that sufficient resources are made available. We also assume that an implemented course with a larger student cohort would be well received. Therefore, future educational research with larger study cohorts will be required to integrate digital health into pharmacy education.

## CRediT authorship contribution statement

**Florian Kinny:** Writing – review & editing, Writing – original draft, Project administration, Methodology, Formal analysis, Data curation, Conceptualization. **Sabina Schlottau:** Writing – review & editing, Writing – original draft, Project administration, Methodology, Formal analysis, Data curation, Conceptualization. **Bushra Ali Sherazi:** Writing – review & editing, Writing – original draft. **Emina Obarcanin:** Writing – review & editing, Supervision, Project administration. **Stephanie Läer:** Writing – review & editing, Supervision, Project administration, Methodology, Conceptualization.

## Declaration of competing interest

The authors declare that they have no known competing financial interests or personal relationships that could have appeared to influence the work reported in this paper.

## Appendix A Supplementary data

Supplementary material 1 Image 1

Supplementary material 2 Image 2
