# Supplementary material for: Digital health in pharmacy education: Elective practical course integrating wearable devices and their generated health data
Source: Explor Res Clin Soc Pharm. 2024 Jun 11;15:100465. doi: 10.1016/j.rcsop.2024.100465 (PMC11231589; doi:10.1016/j.rcsop.2024.100465)
Supplement: Supplementary file 1 — Supplementary material 1 [file mmc1.docx]

| Table A1 |  |
| --- | --- |
| Tuesday, 08.08.2023 09:00 | Tuesday, 08.08.2023 12:00 |
| Breakfast 1 (carbs: 52g)  High-fiber breakfast with berries | Lunch 1 (carbs: 30g)  Healthy Salad |
| - 100 g strawberries - 1 carrot - 100 g cucumber - 80 cherry tomatoes - 150 g natural yogurt - 4 tbsp spelt flakes - 2 tbsp ground linseed - Orange juice | • Lettuce  • Tomato  • Cucumber  • Corn  • Bell pepper  • Chicken  • Balsamic dressing  • Water |
| Wednesday, 09.08.2023 09:00 | Wednesday, 09.08.2023 12:00 |
| Breakfast 2 (carbs: 32g)  Breakfast with berries | Lunch 2 (carbs: 92g)  Cafeteria-meal |
| - 100 g strawberries - 1 carrot - 100 g cucumber - 80g cherry tomatoes - 150 g natural yogurt - Orange juice | - 200g potatoes - Carrots - Broccoli - Onions - Leek - Water |
| Thursday, 10.08.2023 09:00 | Thursday, 10.08.2023 12:00 |
| Breakfast 3 (carbs: 28g)  Wholemeal bread with chickpea spread | Lunch 3 (carbs: 116g)  Veggie Buddah Bowl |
| - 50 g chickpeas - 0.5 tbsp lemon juice - 20 g cream cheese - paprika powder, cumin, salt, pepper - 30 g pointed pepper - 1 slice of wholemeal bread - Water | - Basmati rice - Vegetable balls - Cucumbers - Edamame - Carrot - Cashew-peanut mix - Dark Teriyaki-sauce - Water |
| Friday, 11.08.2023 09:00 | Friday, 11.08.2023 12:00 |
| Breakfast 4 (carbs: 22g)  White bread with chickpea spread | Lunch 4 (carbs: 7g)  Low-Carb – Zoodles with pesto |
| - 50 g chickpeas - 0.5 tbsp lemon juice - 20 g cream cheese - paprika powder, cumin, salt, pepper - 30 g pointed pepper - 1 slice of white bread - Water | - Zoodles (noddles made of zucchinis) - Cherry tomatoes - Pesto - Parmesan - Water |
| Monday, 14.08.2023 09:00 | Monday, 14.08.2023 13:00 |
| Breakfast 5 (carbs: 84g)  Cheat Day | Lunch 5 (carbs: 60g)  Cheat Day |
| - scrambled eggs - Croissant - Nutella - Marmelade - Orange juice | - Fries - Gravy sauce - Mozarella - apple juice |

| Table A2 |  |
| --- | --- |
| Brisk walking | Running |
| - 3.8 kilometers - 45 minutes | - 5 kilometers - 20 minutes |
| Weight exercises in the gym  (each exercise is repeated twice, 30 second-break after each repetition and exercise) | |
| 1. Leg press  - 50 kg - 10 repetitions | 1. Lat pull-down machine  - 35 kg - 12 repetitions |
| 1. Chest press  - 10 kg - 12 repetitions | 1. Rowing machine  - 20 kg - 12 repetitions |
| 1. Shoulder press  - 13 kg - 12 repetitions | 1. Lower back exercise  - No extra weight - 12 repetitions |
| 1. Crunches  - 14 kg - 12 repetitions |  |
| Functional Fitness  (30 second-break after each repetition and exercise) | |
| 1. Jumping Jacks  - 40 seconds | 1. Raising knees, rotate from the inside to the outside or vice versa  - 12x each knee |
| 1. Heels up  - 20 seconds each side | 1. Lunge  - Place legs shoulder-width apart - Leg out at a 90° angle - Knee must not point over toes - Step back to starting position - Then with other leg - 12 repetitions |
| 1. Sit-ups  - Supine position with bent legs - Arms to the temples or chest - Do not pull on the head) - Exhale when going up - Breathe in as you go down - Lower back remains on the floor - 2x 15 repetitions | 1. Super(wo)man  - Lie on your stomach - Arms stretched out in front - Raise arms and legs & wiggle - 2x 40 seconds |
| 1. Squat Jumps  - Legs slightly wider than shoulder width - Start with a squat - Do a jump from this position - Note: Always bend your knees over your ankles, not inwards - 2x 12 repetitions | 1. Planks  - Lean on toes and elbows - Back straight - Buttocks remain in line with shoulders and heels - Hold for 2x 40 seconds |
| 1. Hip Thrust  - Lie on your back - Bend legs, arms outstretched from body - From this position, push the pelvis upwards - Hold in the top position for three seconds - 2x 12 repetitions | 1. Four-footed leg raise  - Get into the four-footed position - Weight is distributed on the knees and hands - Knees under hips, hands under shoulders - Raise your leg and point the sole towards the ceiling - Rock in the top position for 30 seconds - Alternate each leg twice |
| 1. Push-ups  - Sit on your hands and knees - Tense your core - Knees, buttocks, stomach & shoulders form a line - Exhale while pushing up - 2x 8 - 12 repetitions | 1. Rowing with a water bottle  - Slightly bent legs - Wide stance - Lean forward with a straight back - Hold filled bottle in one hand - Raise both arms, pressing shoulder blades together - Lower arms, transfer bottle to other hand - 2x 15 repetitions |
| 1. Four-footed stance  - Get into the four-footed stance - Weight is distributed on the knees and hands - Knees under hips, hands under shoulders - Extend arm and opposite leg - Then pull your elbow towards your knee, making a hump - Then switch sides - 2x 12 repetitions |  |

| Table A3 |  |
| --- | --- |
| **Stress test** Number: __________________  The test consists of 20 topic blocks.  You have three minutes for each of the blocks. MemoryMemorise words You now have one minute to memorise the following 20 words.   \| Number \| Clock \| \| --- \| --- \| \| Head \| Selfie \| \| Water \| Canister \| \| Mouse \| Step \| \| Box \| Semester \| \| Bucket \| Cable \| \| Book \| House \| \| Photo \| Cinema \| \| Bus \| Donkey \| \| Laptop \| Bag \|   Write down all the words you have memorised.  You have two minutes to do this. The order does not matter.   \| 1 \|  \| \| --- \| --- \| \| 2 \|  \| \| 3 \|  \| \| 4 \|  \| \| 5 \|  \| \| 6 \|  \| \| 7 \|  \| \| 8 \|  \| \| 9 \|  \| \| 10 \|  \| \| 11 \|  \| \| 12 \|  \| \| 13 \|  \| \| 14 \|  \| \| 15 \|  \| \| 16 \|  \| \| 17 \|  \| \| 18 \|  \| \| 19 \|  \| \| 20 \|  \|  General knowledgeExtend sentences You will be given sentences to complete with one of the solution words a-d.  Only one of the solution words is correct.   \| 1 \| The symbol ∑ stands for … \| \| \| --- \| --- \| --- \| \|  \| a Energy \| b Square root \| \|  \| c Sum \| d Element \|  \| 2 \| … is no mammal. \| \| \| --- \| --- \| --- \| \|  \| a Cat shark \| b Dolphin \| \|  \| c Seal \| d Whale \|  \| 3 \| 10 centimetres correspond to … millimetres. \| \| \| --- \| --- \| --- \| \|  \| a 10 \| b 100 \| \|  \| c 1000 \| d 10000 \|  \| 4 \| Closest to the sun is … \| \| \| --- \| --- \| --- \| \|  \| a the Earth \| b the Moon \| \|  \| c the planet Mercury \| d the planet Mars \|  \| 5 \| A deficiency of ... can favour or trigger rickets. \| \| \| --- \| --- \| --- \| \|  \| a Vitamin A \| b Vitamin C \| \|  \| c Vitamin D \| d Vitamin E \|  \| 6 \| An ordinary guitar has … strings. \| \| \| --- \| --- \| --- \| \|  \| a 4 \| b 6 \| \|  \| c 8 \| d 10 \|  \| 7 \| Sigmund Freud established the term … for the conscience. \| \| \| --- \| --- \| --- \| \|  \| a “Gesellschafts-Ich” \| b “Über-Ich” \| \|  \| c “Gewissens-Ich” \| d “Gruppen-Ich“ \|  \| 8 \| "On the Origin of Species" was published in 1859 by ... \| \| \| --- \| --- \| --- \| \|  \| a Charles Darwin \| b Gregor Mendel \| \|  \| c Konrad Lorenz \| d Jean-Baptiste de Lamarck \|  \| 9 \| The Titanic sank … \| \| \| --- \| --- \| --- \| \|  \| a 1900 \| b 1912 \| \|  \| c 1920 \| d 1945 \|  \| 10 \| The sign for infinite is ... \| \| \| --- \| --- \| --- \| \|  \| a O \| b ø \| \|  \| c ∆ \| d ∞ \|  Pharmacokinetics Calculate the following tasks for the given patient.  A patient (43 years old, 1.95 m, 100 kg) has been admitted to the hospital where you work due to severe pneumonia. The doctor is planning intravenous therapy with teicoplanin and asks for advice on the dosage.  Elimination constant (k_e_) = 0,02/h  Volume of distribution (V_d_) = 80l  For successful therapy, the trough level should be >10mg/l.   \| 1 \| What is the half-life of teicoplanin?  Hint: ln(2) = 0,7 \| \| --- \| --- \| \| 2 \| When is teicoplanin considered completely eliminated? \| \| 3 \| What is the total body clearance (CL)? \| \| 4 \| The doctor informs you that an intravenous bolus should be given first and then an intravenous infusion (over 48 hours).  The target concentration is 15 mg/l.  Calculate the saturation dose ...  ... and the maintenance dose. \|  LogicWeekdays The task is to determine the logically correct day of the week.   \| 1 \| Tomorrow is four days until Sunday.  What day is the day after tomorrow?  Answer: \| \| --- \| --- \|  \| 2 \| The day after tomorrow is the third day after Monday.  Which day was yesterday?  Answer: \| \| --- \| --- \|  \| 3 \| The day before yesterday is three days after Saturday.  So today is?  Answer: \| \| --- \| --- \|  Spatial thinkingFinding figures The shapes in the top box reappear in the bottom box, but with numbers instead of letters. Assign a shape with a number to each shape with a letter.   \| 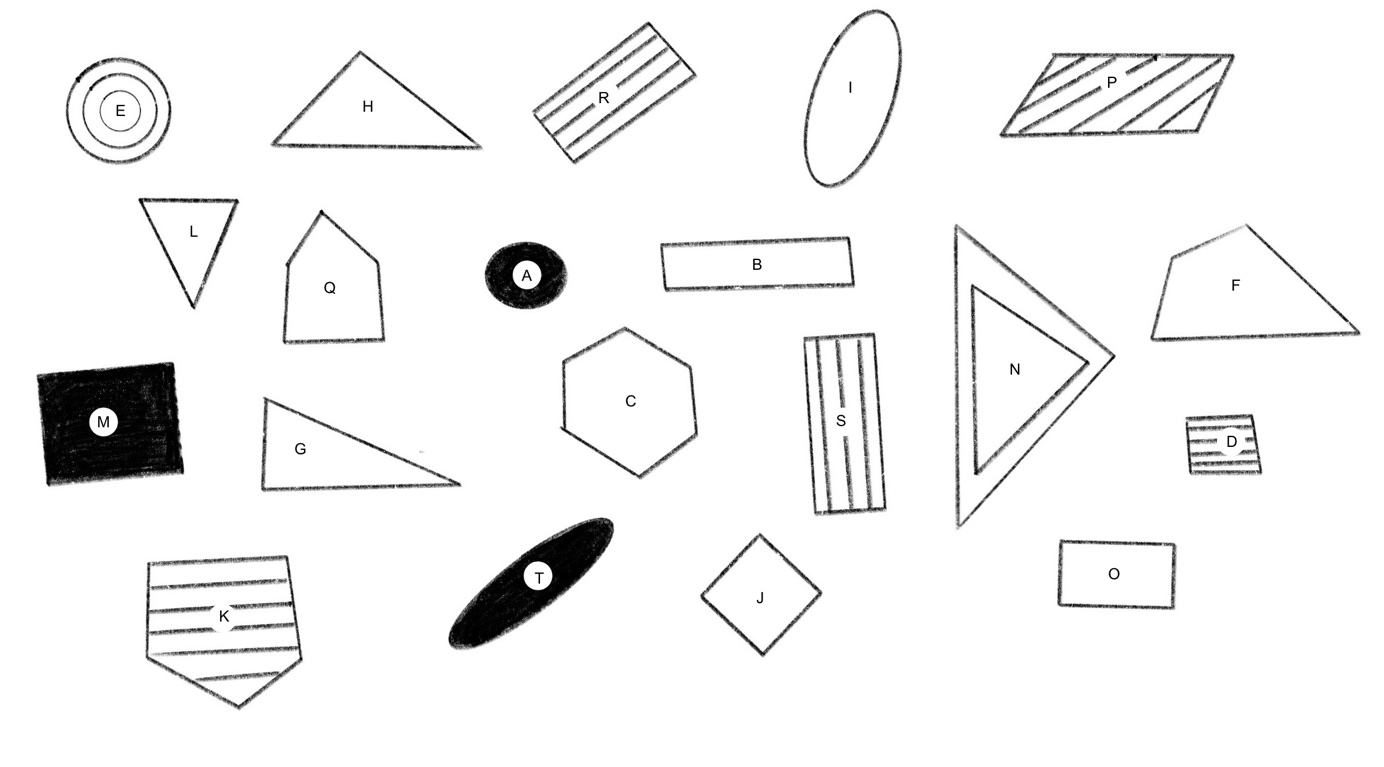 \| \| --- \| \| 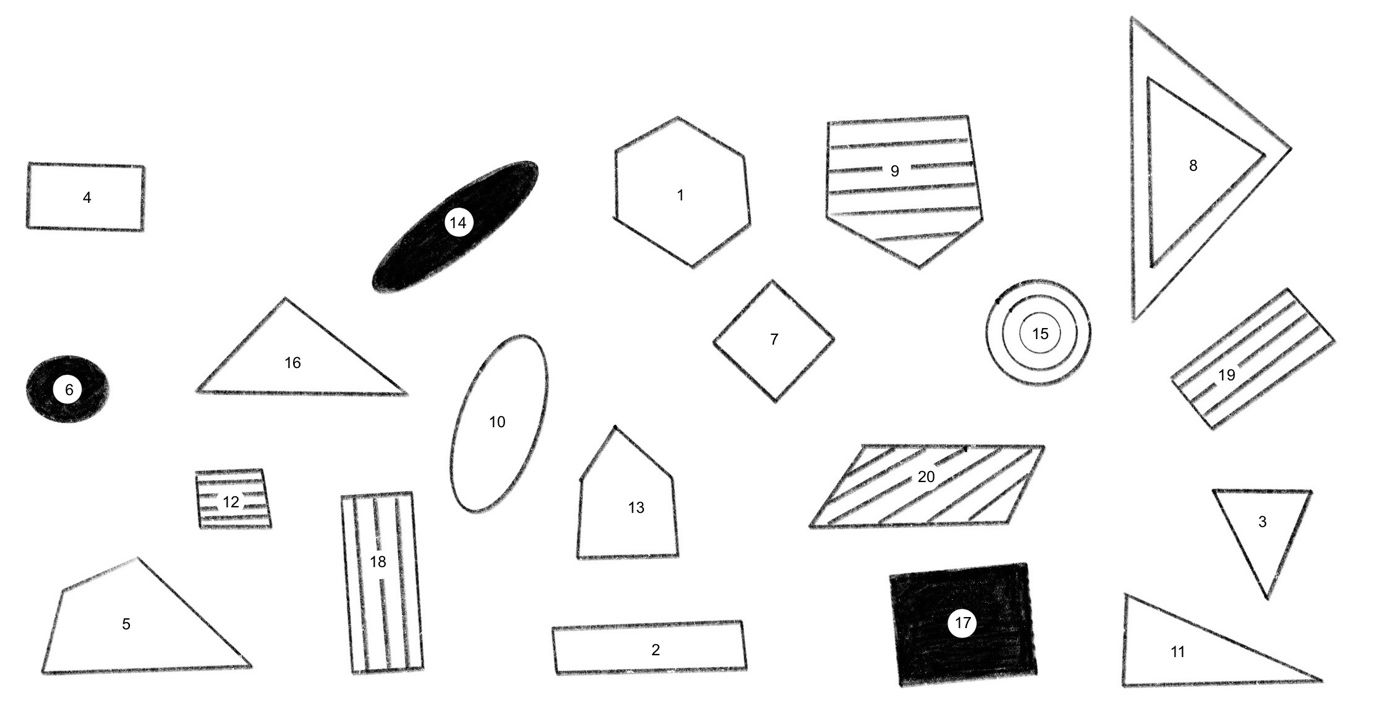 \| \| Answer: \|  Science Answer the following questions using the solution words a-d. Only one solution word is correct for each question.   \| 1 \| What substance is organic chemistry based on? \| \| \| --- \| --- \| --- \| \|  \| a Carbon \| b Hydrogen \| \|  \| c Nitrogen \| d Oxygen \|  \| 2 \| How many natural chemical elements are there? \| \| \| --- \| --- \| --- \| \|  \| a 42 \| b 94 \| \|  \| c 100 \| d 114 \|  \| 3 \| What is the correct chemical name for this drug according to IUPAC? 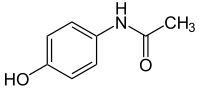 \| \| \| --- \| --- \| --- \| \|  \| a N-Hydroxyphenylamid \| b N-(4-Hydroxyphenyl)­acetamid \| \|  \| c N-Hydroxy-4-aminophenol \| c ortho-Hydroxyacetanilid \|  \| 4 \| What does the following law state?  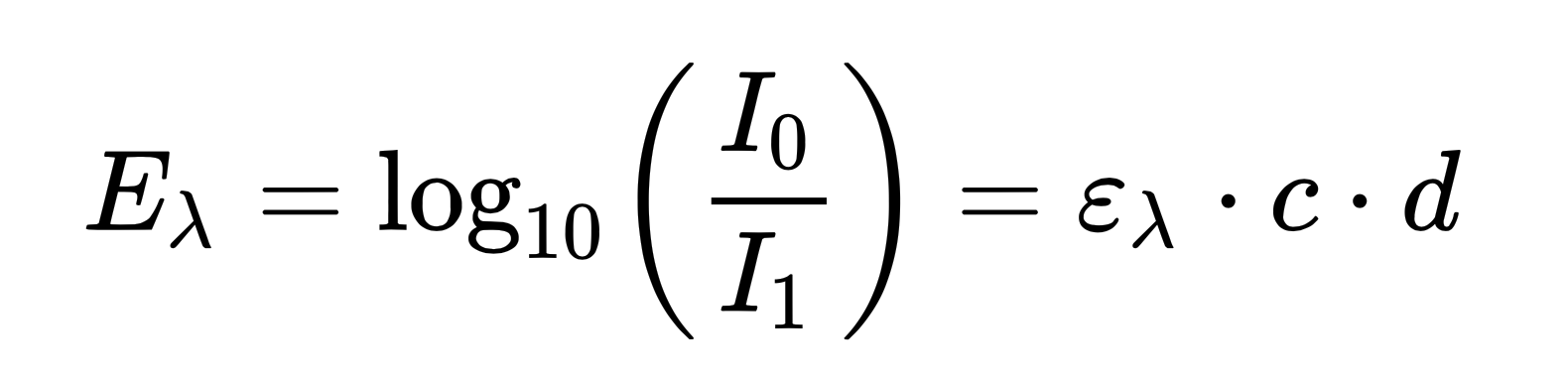 \| \| \| --- \| --- \| --- \| \|  \| a Amplification of the radiation intensity when passing through a medium with an absorbent substance as a function of the concentration of the absorbent substance and the layer thickness \| b The radiation intensity when passing through a medium with an absorbent substance is independent of the concentration of the absorbent substance \| \|  \| c Attenuation of the radiation intensity when passing through a medium with a reflective substance as a function of the concentration of the reflective substance and the layer thickness \| d Attenuation of the radiation intensity when passing through a medium with an absorbent substance as a function of the concentration of the absorbent substance and the layer thickness \|  \| 5 \| Which of the following methods is often used to determine the pH value of a solution? \| \| \| --- \| --- \| --- \| \|  \| a Polarimetry \| b IR spectroskopy \| \|  \| c Potentiometry \| c Chromatography \|  Mathematics Solve the following maths problems and choose from the possible solutions a-d. Only one solution is correct for each problem.   \| 1 \| 0,04 * 0,02 = ? \| \| \| --- \| --- \| --- \| \|  \| a 0,008 \| b 0,0008 \| \|  \| c 0,006 \| d 0,06 \|  \| 2 \| $\frac{3}{4} \div\frac{1}{5}= ?$ \| \| \| --- \| --- \| --- \| \|  \| a 2 $\frac{1}{4}$ \| b $\frac{4}{9}$ \| \|  \| c 3 $\frac{3}{4}$ \| d 3 $\frac{1}{4}$ \|  \| 3 \| - 4 * 12 = ? \| \| \| --- \| --- \| --- \| \|  \| a 48 \| b - 38 \| \|  \| c - 48 \| d 0 \|  \| 4 \| 14 * 8 = 7* ? \| \| \| --- \| --- \| --- \| \|  \| a 15 \| b 16 \| \|  \| c 18 \| d 19 \|  \| 5 \| $\frac{2}{3}+ \frac{1}{2}= ?$ \| \| \| --- \| --- \| --- \| \|  \| a $\frac{7}{6}$ \| b 3 $\frac{3}{8}$ \| \|  \| c $\frac{3}{4}$ \| d 1 $\frac{1}{3}$ \|  ConcentrationAdd letters One letter is missing from each of the following words. Fill in the missing letter.  (German words were chosen for the test because the course was held in German. To minimise distortions, the words in this task were not translated.)   \| 1 \| Au_o \|  \| 51 \| Ap_theke \| \| --- \| --- \| --- \| --- \| --- \| \| 2 \| Schrei_tisch \|  \| 52 \| Kr_ativität \| \| 3 \| Co_puter \|  \| 53 \| Pfo_e \| \| 4 \| Fra_ebogen \|  \| 54 \| T_sse \| \| 5 \| K_bel \|  \| 55 \| Stu_ierende \| \| 6 \| F_asche \|  \| 56 \| Ax_ \| \| 7 \| Ba_k \|  \| 57 \| D_ch \| \| 8 \| Fr_sur \|  \| 58 \| Fa_rrad \| \| 9 \| Mu_ik \|  \| 59 \| N_del \| \| 10 \| _oto \|  \| 60 \| So_ne \| \| 11 \| Kart_n \|  \| 61 \| Phy_iologie \| \| 12 \| D_se \|  \| 62 \| Ru_ksack \| \| 13 \| T_te \|  \| 63 \| Ent_ \| \| 14 \| Bal_on \|  \| 64 \| O_ean \| \| 15 \| Ta_tatur \|  \| 65 \| M_tze \| \| 16 \| _aus \|  \| 66 \| Uh_ \| \| 17 \| Ra_ete \|  \| 67 \| Pi_guin \| \| 18 \| _ampe \|  \| 68 \| Käs_ \| \| 19 \| M_il \|  \| 69 \| Küh_schrank \| \| 20 \| Te_efon \|  \| 70 \| Gl_s \| \| 21 \| Progr_mm \|  \| 71 \| B_nane \| \| 22 \| Bu_h \|  \| 72 \| _ule \| \| 23 \| Kuc_en \|  \| 73 \| Pap_er \| \| 24 \| Pi_za \|  \| 74 \| La_pe \| \| 25 \| Sp_lmaschine \|  \| 75 \| Sch_üssel \| \| 26 \| Bilde_rahmen \|  \| 76 \| Mo_d \| \| 27 \| S_ort \|  \| 77 \| Gi_arre \| \| 28 \| Ar_t \|  \| 78 \| Rett_ngswagen \| \| 29 \| P_lanze \|  \| 79 \| Po_mes \| \| 30 \| Gemäl_e \|  \| 80 \| _ose \| \| 31 \| _alat \|  \| 81 \| Del_in \| \| 32 \| Te_t \|  \| 82 \| Inse_ten \| \| 33 \| Sch_ff \|  \| 83 \| Fe_erball \| \| 34 \| Ve_such \|  \| 84 \| Ap_ikose \| \| 35 \| Druc_er \|  \| 85 \| Quitt_ng \| \| 36 \| _obby \|  \| 86 \| Tie_ \| \| 37 \| M_cke \|  \| 87 \| Au_or \| \| 38 \| La_erne \|  \| 88 \| B_ckerei \| \| 39 \| Kakt_s \|  \| 89 \| Was_hmittel \| \| 40 \| _eans \|  \| 90 \| Anal_tik \| \| 41 \| Vo_el \|  \| 91 \| Nus_ \| \| 42 \| Wa_ser \|  \| 92 \| Universi_ät \| \| 43 \| Ora_ge \|  \| 93 \| A_fel \| \| 44 \| _rzneimittel \|  \| 94 \| Gir_ffe \| \| 45 \| Mu_eum \|  \| 95 \| Ma_melade \| \| 46 \| Hi_mel \|  \| 96 \| Jog_inghose \| \| 47 \| Bal_ \|  \| 97 \| Na_horn \| \| 48 \| W_nd \|  \| 98 \| Papr_ka \| \| 49 \| Sch_h \|  \| 99 \| Qua_k \| \| 50 \| Qu_lle \|  \| 100 \| En_e \|  PharmakotherapyComplete tables Fill in the missing terms.   \| Cardinal symptoms of inflammation \| \| \| \| \| \| --- \| --- \| --- \| --- \| --- \| \|  \| Pain \|  \| Flush \|  \| \| Functio laesa \|  \|  \|  \| Calor \|  \| Infections of the urogenital tract \| \| \| --- \| --- \| \| **Infection** \| **Symptoms** \| \| Pyelonephritis \|  \| \|  \|  \| \|  \| Pain in the urethra, alguria, pollakiuria \|  KreativityFinding words You will see six initial letters, for each of which you should write down six nouns that immediately come into your mind.   \| Initial letter **G** \| Initial letter **A** \| Initial letter **U** \| \| --- \| --- \| --- \| \| G \| A \| U \| \| G \| A \| U \| \| G \| A \| U \| \| G \| A \| U \| \| G \| A \| U \| \| G \| A \| U \|  \| Initial letter **W** \| Initial letter **K** \| Initial letter **P** \| \| --- \| --- \| --- \| \| W \| K \| P \| \| W \| K \| P \| \| W \| K \| P \| \| W \| K \| P \| \| W \| K \| P \| \| W \| K \| P \|  Memory IIMemorise numbers You now have one minute to memorise the following 12 numbers.  24 44 21 85 72 75  63 32 12 19 97 84  Now write down all the numbers you have memorised.  You have two minutes to do this. The order does not matter.   \| 1 \|  \|  \| 4 \|  \|  \| 7 \|  \|  \| 10 \|  \|  \| \| --- \| --- \| --- \| --- \| --- \| --- \| --- \| --- \| --- \| --- \| --- \| --- \| \| 2 \|  \|  \| 5 \|  \|  \| 8 \|  \|  \| 11 \|  \|  \| \| 3 \|  \|  \| 6 \|  \|  \| 9 \|  \|  \| 12 \|  \|  \|  General knowledge II Answer the following questions using the solution words a-d. Only one solution word is correct for each question.   \| 1 \| What does the so-called placebo effect of a medication refer to? \| \| \| --- \| --- \| --- \| \|  \| a Phantom pain \| b a pseudo drug \| \|  \| c a physical effect \| d a clinical trial drug \|  \| 2 \| How many federal states does Germany have? \| \| \| --- \| --- \| --- \| \|  \| a 12 \| b 15 \| \|  \| c 16 \| d 18 \|  \| 3 \| The most expensive are 100 grams ... \| \| \| --- \| --- \| --- \| \|  \| a Silver \| b Gold \| \|  \| c Platinum \| d Titanium \|  \| 4 \| The Louvre Museum is located in ... \| \| \| --- \| --- \| --- \| \|  \| a London \| b Marseille \| \|  \| c Madrid \| d Paris \|  \| 5 \| BND is an abbreviation for ... \| \| \| --- \| --- \| --- \| \|  \| a “Bundesnachrichtendienst” \| b“Bundesverband Natur Deutschland” \| \|  \| c “Bundesamt für Nationales “ \| d “Bürokratendienst” \|  \| 6 \| Which substance do most fertilisers contain? \| \| \| --- \| --- \| --- \| \|  \| a Carbon \| b Oxygen \| \|  \| c Nitrogen \| d Hydrogen \|  \| 7 \| How many planets belong to our solar system? \| \| \| --- \| --- \| --- \| \|  \| a 5 \| b 7 \| \|  \| c 8 \| d 10 \|  \| 8 \| How old is the earth approximately? \| \| \| --- \| --- \| --- \| \|  \| a 1 million years \| b 10 million years \| \|  \| c 100 million years \| d 4,5 billion years \|  \| 9 \| What is usually meant by "www"? \| \| \| --- \| --- \| --- \| \|  \| a “Wieso-Weshalb-Warum” \| b “World Wide Web” \| \|  \| c “Welt-Weite-Wirtschaft” \| d “World Wide Weather” \|  \| 10 \| What do you call a mostly educational story with content from the animal world? \| \| \| --- \| --- \| --- \| \|  \| a Comedy \| b Drama \| \|  \| c Fable \| d Legend \|  Pharmakokinetics II Complete the following graphics.   \| 1 \| 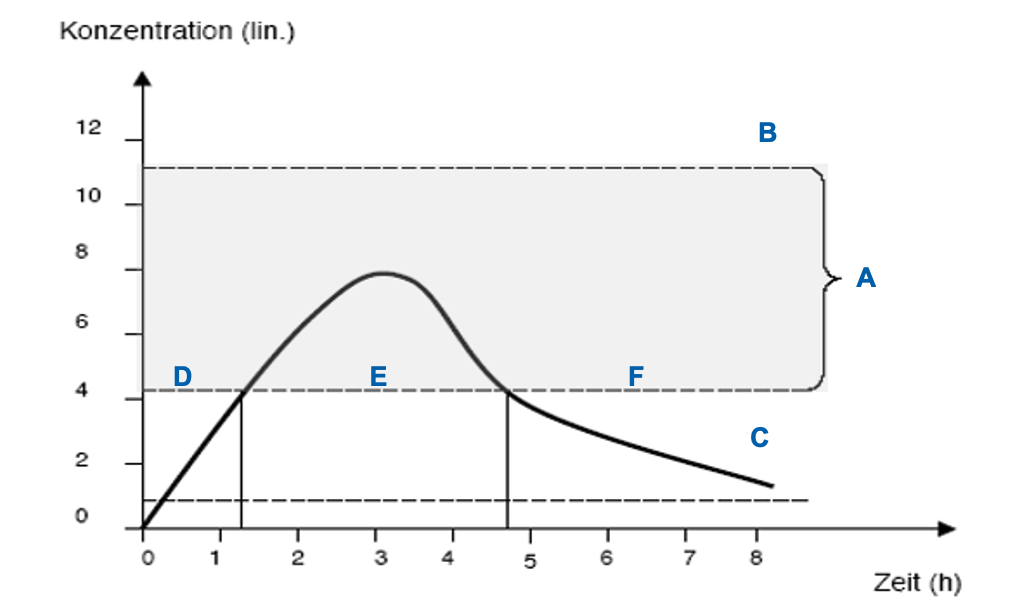  Time (h)  concentration  A:  B:  C:  D:  E:  F: \| \| --- \| --- \|  \| 2 \| 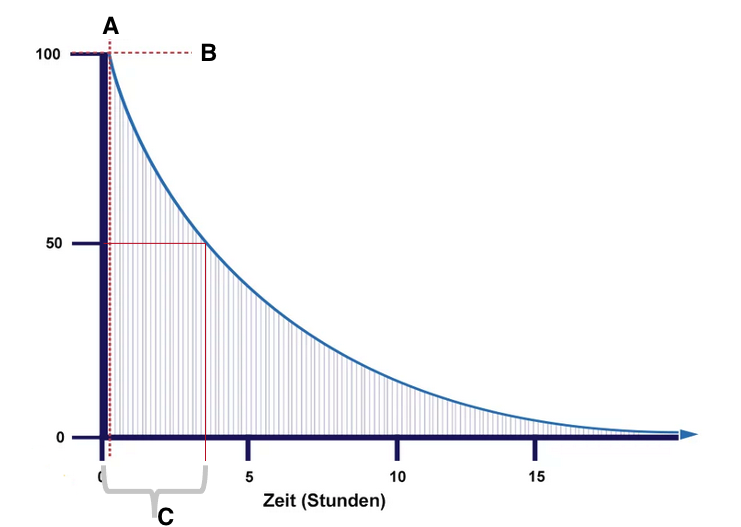  Time (h)  A:  B:  C: \| \| --- \| --- \|  Logics II Your task is to select the word from the proposed solutions a-d that meaningfully completes the equation.  Example: Straight line/ square = curve/ circle   \| 1 \| Sounds/ music = words/ ... \| \| \| --- \| --- \| --- \| \|  \| a Voices \| b Language \| \|  \| c Sounds \| d Expression \|  \| 2 \| Joy/ success = tiredness/ ... \| \| \| --- \| --- \| --- \| \|  \| a Work \| b Dream \| \|  \| c Break \| d Journey \|  \| 3 \| Diet/ weight = medication/ ... \| \| \| --- \| --- \| --- \| \|  \| a Physician \| b Prescription \| \|  \| c Patience \| d Illness \|  \| 4 \| Flood/ dam = rain/ ... \| \| \| --- \| --- \| --- \| \|  \| a Drops \| b Water \| \|  \| c Umbrella \| d Cold \|  Spatial thinking IIFolding figures For each task, you will see a folding template and four figures. Which of the figures can be formed from the folding template (outside)?   \| 1 \| 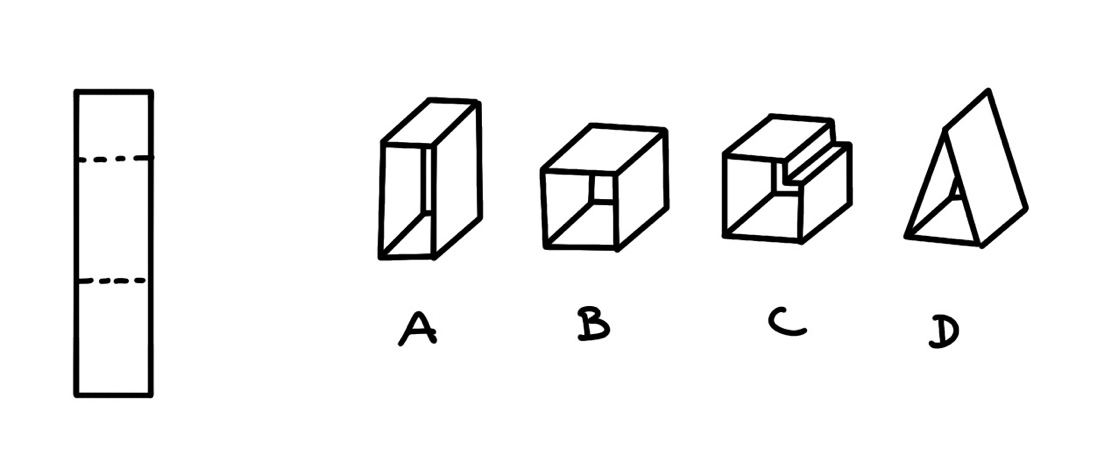  Answer: \| \| --- \| --- \|  \| 2 \| 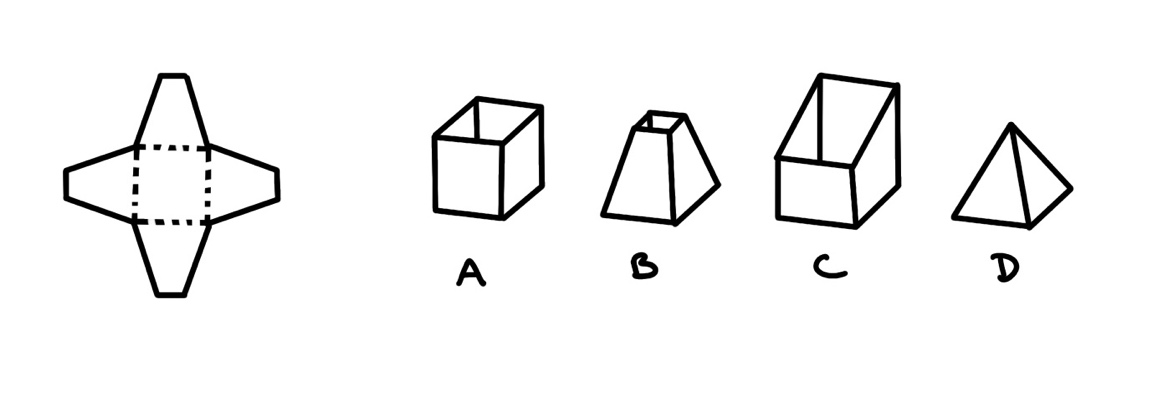  Answer: \| \| --- \| --- \|  \| 3 \| 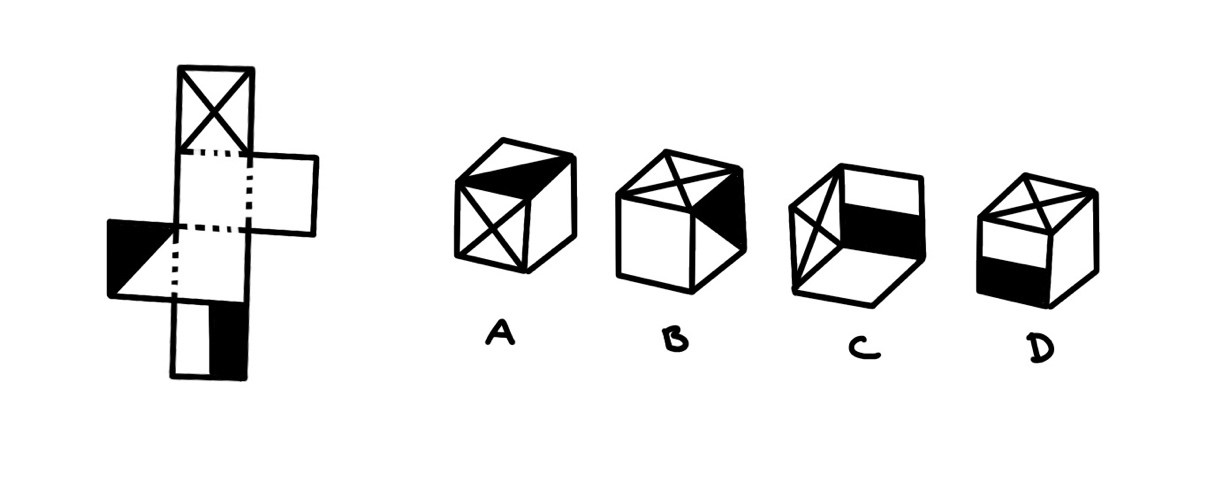  Answer: \| \| --- \| --- \|  \| 4 \| 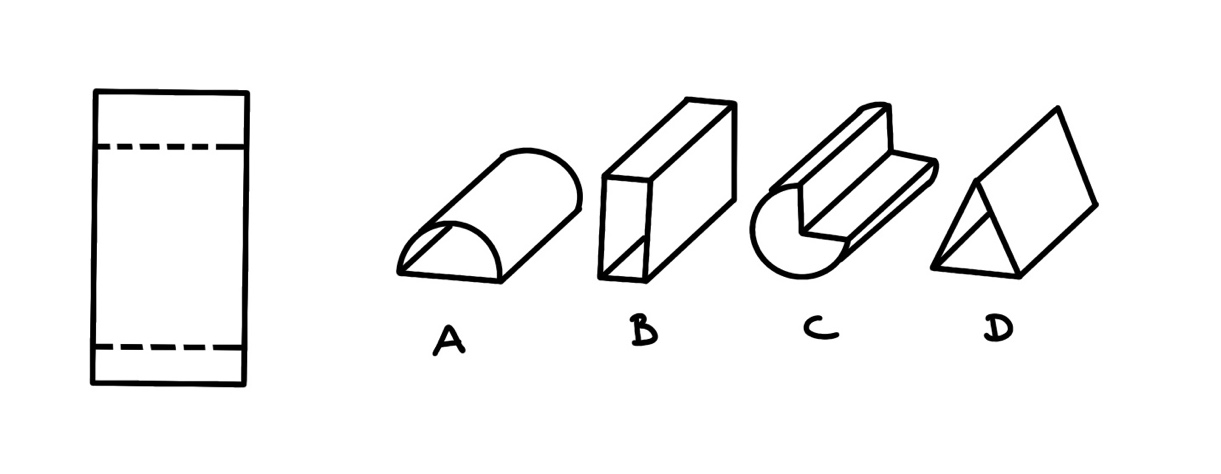  Answer: \| \| --- \| --- \|  Science II Answer the following questions using the solution words a-d. Only one solution word is correct for each question.   \| 1 \| Which of the following amino acids is not an essential amino acid? \| \| \| --- \| --- \| --- \| \|  \| a Lysine \| b Methionine \| \|  \| c Alanine \| d Phenylalanine \|  \| 2 \| Which statement about DNA is true? \| \| \| --- \| --- \| --- \| \|  \| a DNA contains the four nucleobases adenine, cytosine, guanine and uridine. \| b The nucleotides consist of three building blocks: Nucleobase, deoxyribose and phosphate. \| \|  \| c The basic structure of DNA consists of ribose. \| d In eukaryotic cells, the DNA is located freely in the cytosol. \|  \| 3 \| At which temperature is absolute zero? \| \| \| --- \| --- \| --- \| \|  \| a at 0 °C \| b at approx. -100 °C \| \|  \| c at approx. -273 °C \| d at approx. -254 °C \|  \| 4 \| Which statement about cell division is true? \| \| \| --- \| --- \| --- \| \|  \| a There are two different forms of cell division: mitosis and meiosis \| b Gametes (germ cells) are formed during mitosis. \| \|  \| c Mitosis produces two daughter cells whose DNA differs from the DNA of the mother cell. \| d Mitosis consists of prophase, metaphase and anaphase. \|  \| 5 \| When does litmus paper change colour to red? \| \| \| --- \| --- \| --- \| \|  \| a in contact with acid \| b in contact with bases \| \|  \| c in contact with oxygen \| c in contact with neutral solutions \|  Mathematics II Solve the following two text exercises.  1 Mrs Flower has bought new plants for her balcony. She has planted four 80 cm long boxes with five petunias each and three 60 cm long boxes with four begonias each.  One plant costs €1.50. How much money did Mrs Flower pay for her plants?  Answer:  2 You want to buy a carpet. If 4 $\frac{1}{2}$ metres of carpet cost 90€, how much does 2 $\frac{1}{2}$ metres cost?  Answer: Concentration IICounting letters You will see five letter sequences and the solution fields on the right.  Count and enter how often the letters "A", "C", "N" and "T" appear in the following sequences.   \|  \|  \| A \| C \| N \| T \| \| --- \| --- \| --- \| --- \| --- \| --- \| \| 1 \| H S C L N R A F B Q O W D A V C Y F J P W R T L I U C T D K \|  \|  \|  \|  \| \| 2 \| X C K E G T V I P M Y R C O N H Z Q F G B M A K Z P Q A N P \|  \|  \|  \|  \| \| 3 \| J T M Q Z D V L R G A P Y W K S X M L O A X L J D N K G W I \|  \|  \|  \|  \| \| 4 \| T U I O B L S C N M P R C F X G B R W A O C Ö J O A V B I M \|  \|  \|  \|  \| \| 5 \| A K P Y G C L N W D S A H T X B L O S P C T Z U O D S P Q Y \|  \|  \|  \|  \|  Pharmacotherapy II Answer the following questions using the solution words a-d. Only one solution word is correct for each question.   \| 1 \| Which statement about pain is true? \| \| \| --- \| --- \| --- \| \|  \| a A distinction can be made between two different pain fibres (A∂ and C), whereby A∂ fibres conduct pain faster than C fibres. \| b Pain fibres are all myelinated, which gives them a high conduction velocity. \| \|  \| c Chronic pain is not an indication for pain therapy. \| d When using opioid analgesics, both potency and side effect profile must be taken into account, whereas NSAIDs can always be used without hesitation. \|  \| 2 \| Various drug classes can be used in the treatment of unipolar depression. Which drug class is typically NOT used in the treatment of unipolar depression? \| \| \| --- \| --- \| --- \| \|  \| a SSRI (e.g. fluoxetine) \| b Phytopharmaceuticals (e.g. Hypericum perforatum) \| \|  \| c TCA (e.g. amitriptyline) \| d MAO-B inhibitors (e.g. rasagiline) \|  \| 3 \| Which clinical picture are absences, focal seizures and generalised seizures assigned to? \| \| \| --- \| --- \| --- \| \|  \| a Schizophrenia \| b Epilepsy \| \|  \| c Borderline \| d Bipolar disorder \|  \| 4 \| Which statement about sleep is true? \| \| \| --- \| --- \| --- \| \|  \| a Sleep is influenced by hormonal processes such as melatonin, which is a hormone that keeps us awake. \| b Short-term sleep disorders can be managed by improving sleep hygiene, while long-term sleep disorders always require the use of hypnotics. \| \|  \| c There are different stages of sleep, which can be divided into REM sleep and non-REM sleep. \| d The sleep-wake rhythms of young and old people hardly differ. \|  \| 5 \| Which disease is caused by a dopamine deficiency and manifests itself in particular through motor symptoms? \| \| \| --- \| --- \| --- \| \|  \| a Epilepsy \| b Parkinson's syndrome \| \|  \| c Vascular dementia \| d Alzheimer's disease \|  Creativity IIDrawing figures You have 16 circles and 16 squares at your disposal, from which you should draw different real objects or symbols. It's not about the beauty of the graphics, but about ingenuity and variety.   \|  \|  \|  \|  \| \| --- \| --- \| --- \| --- \| \|  \|  \|  \|  \| \|  \|  \|  \|  \| \|  \|  \|  \|  \|  \|  \|  \|  \|  \| \| --- \| --- \| --- \| --- \| \|  \|  \|  \|  \| \|  \|  \|  \|  \| \|  \|  \|  \|  \| | |
