# Supplementary material for: Digital health in pharmacy education: Elective practical course integrating wearable devices and their generated health data
Source: Explor Res Clin Soc Pharm. 2024 Jun 11;15:100465. doi: 10.1016/j.rcsop.2024.100465 (PMC11231589; doi:10.1016/j.rcsop.2024.100465)
Supplement: Supplementary file 2 — Supplementary material 2 [file mmc2.docx]

| **Table A4** | |
| --- | --- |
| Statement | Score (Mean ± Standarddeviation) |
| **Self-Assessment – Pre-Test: Continuous Glucose Monitoring** | |
| How much do you agree with the following statements on the CGM system?  (1=“I don’t agree at all“, 4=“Neutral“, 7=“I agree completely“) | |
| I know the indications for CGM systems | 4.50 (± 0.58) |
| I know the difference between isc and rt CGM systems | 1.75 (± 0.96) |
| I am aware of the advantages of a CGM device compared to SMBG | 3.50 (± 1.91) |
| I am familiar with the structure of a CGM sensor | 3.25 (± 1.71) |
| I know how a CGM sensor performs a glucose measurement | 4.50 (± 0.58) |
| I can explain the principle of "lag-time" | 4.50 (± 1.00) |
| I can explain the term "compression low" | 1.75 (± 0.96) |
| I know what the abbreviation AGP stands for | 1.50 (± 1.00) |
| I know at least ten health metrics | 2.00 (± 2.00) |
| I can recognize the clinical relevance of these health metrics | 3.25 (± 2.63) |
| I can interpret the CGM health metrics | 2.00 (± 1.15) |
| I know the effect of different carbohydrates on the glucose curve | 2.75 (± 0.96) |
| I feel competent enough to apply a CGM system | 2.50 (± 1.29) |
| I feel competent enough to advise people with diabetes on the use and operation of a CGM system | 2.50 (± 1.29) |
| I feel competent enough to interpret CGM data from people with diabetes | 2.25 (± 1.50) |
| I feel competent enough to make treatment recommendations based on CGM data | 2.50 (± 1.91) |
| **Self-Assessment – Pre-Test: Bloodpressure Monitoring** | |
| How much do you agree with the following statements about the blood pressure bracelet?  (1=“I don’t agree at all“, 4=“Neutral“, 7=“I agree completely“) | |
| I know the differences between various blood pressure measurement methods | 3.75 (± 1.71) |
| I can explain the 24-hour blood pressure measurement | 2.00 (± 1.15) |
| I know how an oscillometric blood pressure monitor works | 2.00 (± 0.82) |
| I am familiar with photoplethysmography (PPG) as a technology | 1.25 (± 0.50) |
| I know the contents of the current guidelines on arterial hypertension and type 2 diabetes mellitus. | 5.00 (± 0.82) |
| I am familiar with the blood pressure limits for the diagnosis of arterial hypertension. | 4.25 (± 2.22) |
| I can explain the relevance of arterial hypertension in patients with type 2 diabetes mellitus. | 4.75 (± 2.22) |
| My own blood pressure has already been measured by my doctor | 5.50 (± 1.00) |
| I have already measured my own blood pressure | 5.25 (± 2.36) |
| I have already measured another person's blood pressure | 6.00 (± 1.15) |
| I feel competent enough to provide patient with comprehensive advice on the subject of blood pressure. | 3.25 (± 1.89) |
| I feel qualified to advise polypharmacized patients with type 2 diabetes mellitus and hypertension | 3.00 (± 1.63) |
| I am familiar with the pharmaceutical services (pDL) | 2.50 (± 1.73) |
| I feel qualified to educate patients regarding their blood pressure values | 4.00 (± 1.41) |
| **Self-Assessment – Post-Test: Continuous Glucose Monitoring** | |
|  |  |
| How much do you agree with the following statements on the CGM system?  (1=“I don’t agree at all“, 4=“Neutral“, 7=“I agree completely“) | |
| I know the indications for CGM systems | 6.25 (± 0.50) |
| I know the difference between isc and rt CGM systems | 5.25 (± 1.26) |
| I am aware of the advantages of a CGM device compared to SMBG | 5.75 (± 1.26) |
| I am familiar with the structure of a CGM sensor | 5.25 (± 1.50) |
| I know how a CGM sensor performs a glucose measurement | 6.50 (± 0.58) |
| I can explain the principle of "lag-time" | 5.75 (± 1.50) |
| I can explain the term "compression low" | 4.00 (± 1.41) |
| I know what the abbreviation AGP stands for | 6.50 (± 1.00) |
| I know at least ten health metrics | 5.25 (± 0.96) |
| I can recognize the clinical relevance of these health metrics | 6.25 (± 0.50) |
| I can interpret the CGM health metrics | 5.75 (± 0.50) |
| I know the effect of different carbohydrates on the glucose curve | 5.75 (± 0.96) |
| I feel competent enough to apply a CGM system | 6.75 (± 0.50) |
| I feel competent enough to advise people with diabetes on the use and operation of a CGM system | 6.50 (± 0.58) |
| I feel competent enough to interpret CGM data from people with diabetes | 6.00 (± 0.82) |
| I feel competent enough to make treatment recommendations based on CGM data | 5.50 (± 1.00) |
| **Self-Assessment – Post-Test: Bloodpressure Monitoring** | |
| How much do you agree with the following statements about the blood pressure bracelet?  (1=“I don’t agree at all“, 4=“Neutral“, 7=“I agree completely“) | |
| I know the differences between various blood pressure measurement methods | 5.75 (± 0.96) |
| I can explain the 24-hour blood pressure measurement | 5.50 (± 1.29) |
| I know how an oscillometric blood pressure monitor works | 5.75 (± 0.96) |
| I am familiar with photoplethysmography (PPG) as a technology | 6.00 (± 0.82) |
| I know the contents of the current guidelines on arterial hypertension and type 2 diabetes mellitus. | 6.00 (± 0.82) |
| I am familiar with the blood pressure limits for the diagnosis of arterial hypertension. | 6.25 (± 0.96) |
| I can explain the relevance of arterial hypertension in patients with type 2 diabetes mellitus. | 6.25 (± 0.50) |
| My own blood pressure has already been measured by my doctor | 5.25 (± 1.71) |
| I have already measured my own blood pressure | 6.25 (± 0.96) |
| I have already measured another person's blood pressure | 6.00 (± 2.00) |
| I feel competent enough to provide patient with comprehensive advice on the subject of blood pressure. | 5.75 (± 0.50) |
| I feel qualified to advise polypharmacized patients with type 2 diabetes mellitus and hypertension | 5.00 (± 0.82) |
| I am familiar with the pharmaceutical services (pDL) | 5.50 (± 1.91) |
| I feel qualified to educate patients regarding their blood pressure values | 5.75 (± 0.50) |
| **Assessment: Continuous Glucose Monitoring** | |
| How much do you agree with the following statements on user-friendliness and your satisfaction with the CGM system?  (1=“I don’t agree at all“, 4=“Neutral“, 7=“I agree completely“) | |
| The CGM sensor was easy to apply | 5.75 (± 1.50) |
| The sensor was easy to connect to my mobile device | 5.50 (± 3.00) |
| The CGM system was generally easy to set up | 5.75 (± 0.96) |
| It was easy for me to learn how to use the app | 6.50 (± 0.58) |
| The information in the app was well organized so I could easily find the information I needed | 6.50 (± 0.58) |
| Retrieving the synchronized information in the cloud was easy | 5.30 (± 1.50) |
| The time required to use this app was reasonable for me | 6.50 (± 0.58) |
| Overall. I am satisfied with the CGM system | 6.50 (± 0.58) |
| How useful did you find the various CGM functions for your glucose management?  (1=“Not useful at all“, 5=“Very useful“) | |
| Diary function | 4.75 (± 0.50) |
| Alarm function | 4.75 (± 0.50) |
| Statistics display | 4.75 (± 0.50) |
| Graphics display | 4.75 (± 0.50) |
| Report presentation | 4.75 (± 0.50) |
| Analysis of patterns | 4.50 (± 1.00) |
| Experience. efficiency and effectiveness of the CGM system  (1=“Yes“, 2=“neutral“, 3=“No“) | |
| Have you noticed any improvements in your glucose management since using the CGM system? | 2.00 (± 0.82) |
| Were there any problems or errors that you encountered when using the CGM system? | 2.00 (± 1.15) |
| Has the CGM system helped you to control your glucose levels when traveling or during unexpected events? | 2.00 (± 0.82) |
| Have the functions in the app helped you to deal with hypo- or hyperglycemia? | 2.50 (± 0.58) |
| Has the CGM system helped you to monitor and track trends in your blood glucose levels over time? | 3.00 (± 0.00) |
| Has the CGM system helped you to understand the effects of the type and amount of food you eat? | 2.75 (± 0.50) |
| Has the CGM system helped you to track and manage your carbohydrate intake? | 2.75 (± 0.50) |
| Was it effective to wear a CGM system for two weeks to develop an understanding of the device and glucose levels? | 3.00 (± 0.00) |
| Was it effective to wear a CGM system for two weeks to develop empathy for users? | 3.00 (± 0.00) |
| I felt comfortable using/operating the CGM system in a social environment | 2.75 (± 0.50) |
| **Assessment: Bloodpressure Monitoring** | |
| How much do you agree with the following statements about your satisfaction with the blood pressure bracelet?  (1=“I don’t agree at all“, 4=“Neutral“, 7=“I agree completely“) | |
| The positioning of the wristband went smoothly | 5.00 (± 0.82) |
| The initialization with the upper arm cuff went smoothly | 5.00 (± 0.82) |
| I found wearing the arm band comfortable | 4.75 (± 0.50) |
| The synchronization of the values with the cloud worked without any problems | 5.00 (± 0.82) |
| I was able to get the information I needed from the app. | 5.50 (± 0.58) |
| I feel that the time required to use the app is reasonable | 4.75 (± 1.89) |
| I am satisfied with the bracelet as a blood pressure measurement method | 5.50 (± 0.58) |
| How much do you agree with the following statements on awareness and perception in relation to blood pressure?  (1=“I don’t agree at all“. 4=“Neutral“. 7=“I agree completely“) | |
| I was able to develop an awareness of my blood pressure values | 5.75 (± 0.50) |
| Taking part in the study was an added value for me personally | 5.75 (± 0.50) |
| Blood pressure monitoring has helped me to understand changes in blood pressure | 5.25 (± 0.50) |
| Using a blood pressure monitor and a CGM system has increased my digital competence | 5.50 (± 0.58) |
| I think continuous monitoring of vital signs in patients makes sense | 5.75 (± 0.50) |
| I think continuous monitoring of vital signs in healthy people makes sense | 5.50 (± 0.58) |
| **Assessment: Quality of Life** | |
| In the last two weeks....  (1=“I don’t agree at all“, 6=“I agree completely“) | |
| …I have been happy and in a good mood | 5.00 (± 0.82) |
| …I have felt calm and relaxed | 5.00 (± 0.82) |
| …I have felt energetic and active | 4.00 (± 1.83) |
| …I have woken up feeling fresh and rested | 2.50 (± 2.38) |
| …my everyday life has been full of things that interest me | 5.00 (± 0.82) |
